# Opioid Overdose After Medication for Opioid Use Disorder Initiation Following Hospitalization or ED Visit

**DOI:** 10.1001/jamanetworkopen.2024.23954

**Published:** 2024-07-22

**Authors:** Scott G. Weiner, Kacey Little, Jiah Yoo, Diana P. Flores, Christi Hildebran, Dagan A. Wright, Grant A. Ritter, Sanae El Ibrahimi

**Affiliations:** 1 Department of Emergency Medicine, Brigham and Women’s Hospital, Boston, Massachusetts; 2 Harvard Medical School, Boston, Massachusetts; 3 Comagine Health, Portland, Oregon; 4 Oregon Health Authority, Portland; 5 Brandeis University, Waltham, Massachusetts; 6 School of Public Health, University of Nevada, Las Vegas

## Abstract

**Importance:**

Hospitalizations related to opioid use disorder (OUD) represent an opportunity to initiate medication for OUD (MOUD).

**Objective:**

To assess whether starting MOUD after a hospitalization or emergency department (ED) visit is associated with the odds of fatal and nonfatal opioid overdose at 6 and 12 months.

**Design, Setting, and Participants:**

This population-based cohort study used data from the Oregon Comprehensive Opioid Risk Registry, which links all payer claims data to other administrative health datasets, for individuals aged 18 years or older who had diagnosis codes related to OUD recorded at an index ED visit or hospitalization from January 2017 to December 2019. Data were analyzed between May 2023 and January 2024.

**Exposures:**

Receipt of MOUD within the 7 days after an OUD-related hospital visit.

**Main Outcomes and Measures:**

The primary outcome was fatal or nonfatal overdose at 6 and 12 months after discharge. Sample characteristics, including age, sex, insurance plan, number of comorbidities, and opioid-related overdose events, were stratified by receipt or nonreceipt of MOUD within 7 days after an OUD-related hospital visit. A logistic regression model was used to investigate the association between receipt of MOUD and having an opioid overdose event.

**Results:**

The study included 22 235 patients (53.1% female; 25.0% aged 25-39 years) who had an OUD-related hospital visit during the study period. Overall, 1184 patients (5.3%) received MOUD within 7 days of their ED visit or hospitalization. Of these patients, 683 (57.7%) received buprenorphine, 463 (39.1%) received methadone, and 46 (3.9%) received long-acting injectable naltrexone. Patients who received MOUD within 7 days after discharge had lower adjusted odds of fatal or nonfatal overdose at 6 months compared with those who did not (adjusted odds ratio [AOR], 0.63; 95% CI, 0.41-0.97). At 12 months, there was no difference in adjusted odds of fatal or nonfatal overdose between these groups (AOR, 0.79; 95% CI, 0.58-1.08). Patients had a lower risk of fatal or nonfatal overdose at 6 months associated with buprenorphine use (AOR, 0.50; 95% CI, 0.27-0.95) but not with methadone use (AOR, 0.57; 95% CI, 0.28-1.17).

**Conclusions and Relevance:**

In this cohort study of individuals with an OUD-related hospital visit, initiation of MOUD was associated with reduced odds of opioid-related overdose at 6 months. Hospitals should consider implementing programs and protocols to offer initiation of MOUD to patients with OUD who present for care.

## Key Points

**Question:**

Is starting medication for opioid use disorder (OUD) treatment after a hospital visit associated with lower odds of future fatal or nonfatal opioid overdose?

**Findings:**

In this cohort study of 22 235 patients with an OUD-related hospital visit, 5.3% commenced medication for OUD within 7 days, and these patients had lower adjusted odds of fatal or nonfatal opioid overdose at 6 months.

**Meaning:**

The findings suggest that hospital visits are an opportunity to initiate medication for OUD and are associated with lower odds of opioid overdose at 6 months.

## Teaser

This cohort study assesses the association of medication for opioid use disorder (MOUD) initiation after hospitalization or an emergency department (ED) visit with subsequent fatal or nonfatal opioid overdose at 6 and 12 months.

## Introduction

Opioid-related hospitalizations in the US have increased markedly in the past several years, from 136.8 per 100 000 population in 2005 to 250.4 per 100 000 population in 2020.^1^ Hospitalizations related to opioid use disorder (OUD) comprised about 3.4% of all hospitalizations in metropolitan areas in 2016.^2^ These hospitalizations are not always related to overdose but rather to the spectrum of morbidity that accompanies substance use, including HIV infection, hepatitis, endocarditis, skin and soft tissue infections, and bacteremia.^3^ Opioid-related complications contribute disproportionately to deaths associated with sepsis in younger patients.^4^

Although a hospital visit—either a hospitalization or a visit to the emergency department (ED)—is an undesirable event for any individual, it represents an opportunity to engage patients in medication for OUD (MOUD). Multiple guidelines recommend use of MOUD, specifically buprenorphine, methadone, or naltrexone, to treat OUD and opioid withdrawal in hospitalized patients^5^ and those seen in the ED.^6^ People who initiate buprenorphine treatment during hospitalization experience several improved outcomes, including longer retention in treatment, more drug-free days, and lower odds of readmission.^7,8^ As a result, some hospitals have begun to implement a range of programs, including specialized teams to start buprenorphine treatment in the hospital and creation of general addiction medicine consultation services.^9,10^ In one system, inpatients who received MOUD either on or before the admission date (vs those who did not) were less likely to have unplanned readmission (13% vs 22%),^11^ and another study found that patients engaging with an inpatient addiction consultation service (vs those who did not) had higher rates of substance use disorder treatment following discharge (39% vs 23%).^12^ It is theorized that addiction consultation service expansion would reduce fentanyl-related deaths.^13^ There is evidence of substantial cost savings to the health care system for patients who initiate buprenorphine treatment.^14^

MOUD is underutilized, with approximately 87% of individuals with past-year OUD not receiving it.^15^ Using the hospital visit as an opportunity to start MOUD for patients with OUD could improve this treatment gap. Furthermore, patient-directed discharge is a marked negative outcome for patients with OUD who are hospitalized, particularly for individuals who inject drugs.^16^ A large study evaluating admissions at 362 US hospitals found that administering MOUD in the hospital was associated with a lower risk of patient-directed discharge, yet it was used only 22% of the time for admitted patients with OUD.^17^

The outcomes of starting MOUD following a hospital visit have largely focused on rate of either outpatient follow-up or readmission. For patients not previously receiving MOUD, the association between initiating MOUD following a hospital visit (either ED visit or admission) and a future overdose has gone largely understudied. The aim of this study, therefore, was to address this gap by leveraging a comprehensive linked public health dataset in Oregon that includes ED visits and hospital admissions, prescription drug monitoring program medication dispensations, medication-related billing data, and vital statistics records to assess whether patients diagnosed with OUD who started MOUD after a hospital visit (for reasons other than overdose) had lower adjusted odds for fatal and nonfatal opioid overdose at 6 and 12 months.

## Methods

The Mass General Brigham Human Research Committee approved this cohort study, and patient consent requirement was waived because this study posed minimal risk to participants and the research could not practically be conducted without the waiver. We followed the Strengthening the Reporting of Observational Studies in Epidemiology (STROBE) reporting guideline.

### Data Sources

This retrospective cohort study used a subset of the Oregon Comprehensive Opioid Risk Registry database. The foundational data source for the database is the voluntary Oregon All Payer Claims Database (APCD), which includes Medicaid, Medicare (traditional and Medicare Advantage), and commercial insurance claims for approximately 80% of Oregonians from 2013 to 2020. The APCD was probabilistically linked using first name, last name, and date of birth, with a threshold of 85%, to the Oregon prescription drug monitoring program (PDMP), Oregon vital records death certificates, emergency medical services, and the Oregon hospital discharge database. Discharges from the ED were ascertained from APCD data.^18^

### Study Sample

For the index visit, we identified patients with diagnosis codes related to OUD (*International Statistical Classification of Diseases and Related Health Problems, Tenth Revision [ICD-10]* codes F11.1x-F11.2x and F11.9x) that were recorded at an ED visit or hospitalization from January 2017 to December 2019. We did not specifically evaluate medical diagnosis codes associated with drug use, such as endocarditis, as inclusion criteria, but they could have been present alongside the included codes. We also excluded patients with overdose (poisoning) codes at their index visit, as the intended study population was patients with OUD who had a reason for medical care apart from overdose. When a hospitalization resulted from an ED visit, it was considered a hospitalization and only the final diagnosis codes from the hospitalization were considered. Although we had data on visits predating 2017, we did not include them given the shifting illicit opioid supply that has been more recently dominated by synthetic fentanyl and fentanyl analogues. We included patients aged 18 years or older. Race and ethnicity were not included due to substantial missingness in the data. To ensure capture of all relevant outcomes, we restricted the sample to patients who had continuous insurance enrollment 6 months before and 12 months after the index hospital visit but allowed up to a 60-day gap in coverage. To avoid correlated records, we included only each patient’s first episode in our study period and, thus, excluded individuals with an OUD-related hospitalization or ED visit in the prior 6 months. To assure that we only included patients starting MOUD, we also excluded individuals with evidence of MOUD receipt (either a billing code for clinic-administered buprenorphine, methadone, or naltrexone or a PDMP record of a dispensation of an OUD formulation for buprenorphine) in the previous 6 months (eAppendix 1 in Supplement 1).

### Covariates

We categorized age in groups as of January 1, 2017 (18-24, 25-39, 40-54, 55-74, and ≥75 years). Other covariates included sex, insurance plan type (commercial, Medicaid, Medicare, or dual enrollment), and Elixhauser comorbidity score^19,20^ (0, 1-2, or ≥3 comorbidities) (eAppendix 2 in Supplement 1).

### Exposure

We assessed receipt of MOUD in the 7 days after an OUD-related hospital visit as the main exposure. Use of MOUD was characterized as receipt of buprenorphine, methadone, or naltrexone in an outpatient setting using procedure codes or having a MOUD formulation buprenorphine fill in the PDMP.

### Outcomes

Patients were followed up for 6 and 12 months after their OUD-related hospital visit and assessed for their first occurrence of a nonfatal or fatal opioid-related event. Nonfatal opioid overdose events were captured from hospital discharge records and APCD ED insurance claims for any opioid poisoning events (*ICD-10* codes T40.0, T40.1, T40.2, T40.3, T40.4, and T40.6). Fatal opioid overdose events were captured using underlying cause-of-death codes (*ICD-10* codes X40-X44, X60-X64, X85, and Y10-Y14) with multiple cause-of-death codes (*ICD-10* codes T40.0, T40.1, T40.2, T40.3, T40.4, and T40.6). We also searched the literal text fields of the death record for terms associated with opioid and heroin overdose.^21^ Outcomes were also stratified by MOUD type and location of care (ED visit only vs hospitalization).

### Statistical Analysis

We generated frequency distributions of the sample characteristics, including age, sex, insurance plan, comorbidities, and the opioid-related overdose events, stratified by receipt or nonreceipt of MOUD treatment 7 days after an OUD-related visit discharge. We then used a logistic regression model to investigate the association between receipt of MOUD during that time and having an opioid overdose event within the study’s 6- or 12-month follow-up period. We chose a logistic regression model instead of a Cox proportional hazards regression model because of the qualitative nature of the outcome (an overdose). Our intent was to identify factors associated in any way with outcomes but not factors associated with a later occurrence in the follow-up period. In addition, a logistic regression model does not rely on a proportion hazards assumption. Following our main analysis, we studied patients who received MOUD after an ED visit and then separately those discharged after hospitalization. We also conducted subgroup analyses of each specific agent of MOUD. All secondary analyses adjusted for individual characteristics. Accordingly, these results should be considered exploratory, recommending further follow-up. We conducted all analyses between May 2023 and January 2024 using SAS, version 9.3 (SAS Institute Inc).

## Results

There were 22 235 patients who had an included OUD-related visit during the study period (Figure). Characteristics are displayed in Table 1. The majority were female (53.1%); 46.9% were male. A total of 6.8% were aged 18 to 24 years; 25.0%, 25 to 39 years; 19.3%, 40 to 54 years; 37.0%, 55 to 75 years; and 11.9% 75 years or older. Furthermore, 46 patients (0.2%) had fatal overdose at 6 months, and 76 (0.3%) had fatal overdose at 12 months. The most prevalent insurance types were Medicaid (48.5%) followed by Medicare (28.9%), dual enrollment in Medicaid and Medicare (11.6%), and commercial (7.7%). Most patients (58.4%) had 3 or more Elixhauser comorbidities coded. Medicaid patients had the highest rate of treatment receipt (982 [9.1%]) followed by dually enrolled patients (982 [1.8%]) and Medicare patients (56 [0.9%]) received treatment.

**Figure.**
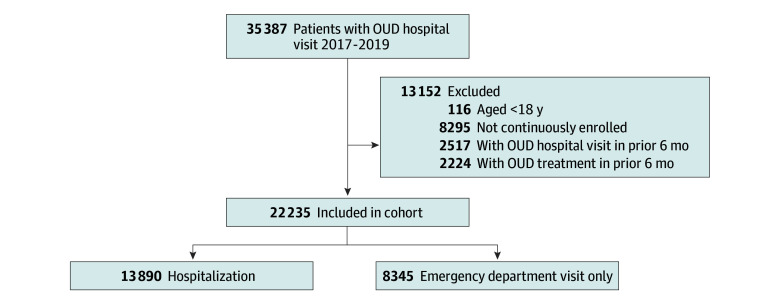
Flow Diagram of Study Patients OUD indicates opioid use disorder.

**Table 1.** Characteristics of Included Patients

Characteristic	Patients, No. (%)		
	Total	Received OUD treatment within 7 d of discharge^a^	
		No	Yes
All	22 235 (100)	21 051 (94.7)	1184 (5.3)
Age, y			
18-24	1516 (6.8)	1356 (89.4)	160 (10.6)
25-39	5557 (25.0)	4936 (88.8)	621 (11.2)
40-54	4302 (19.3)	4058 (94.3)	244 (5.7)
55-74	8221 (37.0)	8071 (98.2)	150 (1.8)
≥75	2639 (11.9)	2630 (99.7)	9 (0.3)
Sex			
Female	11 808 (53.1)	11 180 (94.7)	628 (5.3)
Male	10 426 (46.9)	9870 (94.7)	556 (5.3)
Payer			
Commercial	1709 (7.7)	1614 (94.4)	95 (5.6)
Medicaid	10 786 (48.5)	9804 (90.9)	982 (9.1)
Medicare	6431 (28.9)	6375 (99.1)	56 (0.9)
Dual	2574 (11.6)	2528 (98.2)	46 (1.8)
Unknown	735 (3.3)	730 (99.3)	5 (0.7)
Elixhauser comorbidities, No.			
0	4626 (20.8)	4293 (92.8)	333 (7.2)
1-2	4621 (20.8)	4210 (91.1)	411 (8.9)
≥3	12 988 (58.4)	12 548 (96.6)	440 (3.4)

Abbreviation: OUD, opioid use disorder.

^a^ Row percentages are shown in these columns. Evidence of clinic-administered buprenorphine, methadone, or extended-release naltrexone or prescription drug monitoring program evidence of a filled prescription for a formulation of buprenorphine indicated for OUD.

Overall, 1184 patients (5.3%) received MOUD within 7 days of an ED visit or hospitalization. Of these, 683 (57.7%) received buprenorphine, 463 (39.1%) received methadone, and 46 (3.9%) received long-acting injectable naltrexone. Table 2 shows outcome measures. There were 452 index nonfatal and fatal overdose events in 6 months and 758 overdose events in 12 months. This finding indicates that, of included patients, 2.0% had an overdose event within 6 months and 3.4% had an event within 12 months.

**Table 2.** Overdose Outcomes for the Total Cohort and Stratified by Receipt of OUD Treatment Within 7 Days of Discharge

Outcome	Patients, No. (%)		
	Total (N = 22 235)	Received OUD treatment within 7 d of discharge^a^	
		No (n = 21 051)	Yes (n = 1184)
Fatal or nonfatal overdose			
≤6 mo	452 (2.0)	430 (2.0)	22 (1.9)
≤12 mo	758 (3.4)	712 (3.4)	46 (3.9)
Fatal overdose			
≤6 mo	46 (0.2)	44 (0.2)	2 (0.2)
≤12 mo	76 (0.3)	72 (0.3)	4 (0.3)
Nonfatal overdose			
≤6 mo	411 (1.8)	390 (1.8)	21 (1.8)
≤12 mo	691 (3.1)	648 (3.1)	43 (3.6)

Abbreviation: OUD, opioid use disorder.

^a^ Evidence of clinic-administered buprenorphine, methadone, or extended-release naltrexone or prescription drug monitoring program evidence of a filled prescription for a formulation of buprenorphine indicated for OUD.

The adjusted odds ratios (AORs) of overdose after an OUD-related hospital visit based on receipt of MOUD are given in Table 3. After adjusting for age, sex, payer, and number of comorbidities, patients who received MOUD within 7 days after discharge had lower adjusted odds of fatal or nonfatal overdose at 6 months compared with those who did not (AOR, 0.63; 95% CI, 0.41-0.97). At 12 months, the AOR of fatal or nonfatal overdose was less than 1 but did not reach statistical significance (AOR, 0.79; 95% CI, 0.58-1.08). For fatal overdoses, there were 46 events at 6 months and 76 events at 12 months. There was no association between receipt of MOUD and fatal overdose at 6 months (AOR, 0.63; 95% CI, 0.15-2.66) or 12 months (AOR, 0.73; 95% CI, 0.26-2.02).

**Table 3.** Association of Medication for OUD 7 Days After the Index OUD Hospital Visit With Subsequent 6- and 12-Month Opioid Overdose

Outcome	Adjusted odds ratio (95% CI)	
	6 mo After index OUD event	12 mo After index OUD event
Fatal overdose	0.63 (0.15-2.66)	0.73 (0.26-2.02)
Nonfatal overdose	0.65 (0.42-1.02)	0.81 (0.59-1.11)
Fatal or nonfatal overdose		
Any	0.63 (0.41-0.97)	0.79 (0.58-1.08)
After ED visit only	0.57 (0.33-0.98)	0.85 (0.59-1.21)
After hospitalization only	0.72 (0.35-1.49)	0.59 (0.32-1.10)

Abbreviations: ED, emergency department; OUD, opioid use disorder.

We then separated the cohort into those who received MOUD after an ED visit (eAppendix 3 in Supplement 1) or after discharge after hospitalization (eAppendix 4 in Supplement 1). These results are also shown in Table 3. There were 8345 patients discharged after an ED visit, of whom 694 (8.3%) received MOUD within 7 days of discharge. The fatal or nonfatal overdose rate at 6 months was 2.9% (n = 244) and at 12 months was 5.1% (n = 423). There were lower odds of fatal or nonfatal overdose at 6 months among patients receiving MOUD following an ED visit (AOR, 0.57; 95% CI, 0.33-0.98), but there was no difference in odds at 12 months. There were 13 890 patients discharged after hospitalization, of whom 491 (3.5%) received MOUD within 7 days of discharge. The fatal or nonfatal overdose rate at 6 months was 1.5% (n = 208) and at 12 months was 2.4% (n = 208). Adjusted odds were not significantly different in this subset population.

When evaluating specific agents received, patients had a lower risk of fatal or nonfatal overdose at 6 months associated with buprenorphine use (AOR, 0.50; 95% CI, 0.27-0.95) (eTable 1 in Supplement 1). However, there was no association with methadone use (AOR, 0.57; 95% CI, 0.28-1.17) (eTable 2 in Supplement 1). The AORs for overdose at 12 months with each medication were not significant. Outcomes associated with naltrexone use are not reported due to the small number of events (4 overdoses at 6 months).

## Discussion

This cohort study found that receipt of MOUD within 7 days of a hospital visit was associated with lower odds of fatal or nonfatal overdose at 6 months but not at 12 months. The lower odds of overdose at six months are notable and consistent with previous studies^7,8^ that observed other positive outcomes, such as lower readmission rates and higher retention rates in treatment.

Our study found that only 5.3% of patients had evidence of MOUD initiation, which indicates opportunity for improvement, especially considering another study in Oregon that found that hospitalized patients with OUD had a 7.8% 1-year mortality (4.5% directly from drug-related causes).^22^ Society of Hospital Medicine guidelines^5^ now recommend initiation of MOUD for patients hospitalized with OUD-related issues, and these findings further support that recommendation. We also found that rates varied depending on patient population, with 10.6% of patients aged 18 to 24 years and only 1.8% of those aged 55 to 74 years receiving treatment. These findings are concerning given that OUD and overdose still occur in older populations.^23^ Medicaid patients had the highest rate of treatment receipt (9.1%), but only 1.8% of dually enrolled patients and 0.9% of Medicare patients received treatment. The findings suggest that more interventions are needed to address the broad range of system-level gaps that influence low MOUD prescribing rates in hospitals.^24^

The rate of overdose in the patient population in this study was also of interest. In prior work, 5.5% of ED patients discharged after an overdose died within 12 months.^25^ In the present cohort, only 0.3% of patients had a fatal overdose within 12 months, suggesting that this population may be at less risk than those discharged from an ED after overdose. The former study^25^ occurred in Massachusetts (as opposed to Oregon), and patterns of drug use may differ. Also, the current study captured individuals who had OUD but presented for health care for reasons other than overdose, while the Massachusetts study examined overdose discharges.^25^ Still, the current cohort had a 3.4% risk of fatal or nonfatal overdose at 12 months, which is notable and merits more attention, especially as rates of death are increasing with more synthetic fentanyl analogues in the nonmedical opioid supply.^26,27^ Although the AOR of fatal or nonfatal overdose was lower overall with receipt of MOUD at 6 months, there were also lower odds of fatal or nonfatal overdose at 6 months specifically among patients receiving MOUD after an ED visit, further supporting guidelines that recommend commencing MOUD from the ED setting.^6^

Although MOUD initiation was associated with lower odds of overdose at 6 months, there was no association at 12 months. It is unknown whether this finding is because of the small sample size or a lack of treatment effects. We only evaluated whether MOUD was obtained immediately after hospitalization, but patients were not followed up longitudinally to assess whether they continued receiving MOUD. Retention in treatment is a significant factor associated with decreased mortality,^28^ and further research will need to determine which factors contribute to patients continuing treatment after it is begun in the hospital setting. In, to our knowledge, the first large study of buprenorphine initiation in EDs, there was increased retention in treatment at 30 days, but the associated increase dissipated after 6 months,^29,30^ emphasizing the need for overarching improvements to the OUD care system to make it more patient-centered and accessible.

Buprenorphine use was associated with lower odds of overdose at 6 months, but methadone was not. As with the finding at 12 months, this lack of association could be related to a small number of outcomes. However, it may also represent less effectiveness of methadone started in the hospital setting. Although outcomes associated with methadone and buprenorphine are similar,^31^ there are multiple complexities related to starting methadone in the hospital, including the need to establish linkage to an outpatient treatment program that dispenses methadone. These challenges are not insurmountable, and there is a framework for hospitals to guide implementation.^32,33^ Different than methadone, buprenorphine can be prescribed and dispensed at commercial pharmacies, although access varies. The recent abolishment of the buprenorphine X-waiver may encourage more prescribers to provide this medication to their patients, but barriers at pharmacies remain.^34,35^

The current study examined specifically patient-level factors (ie, age, sex, and number of comorbidities), but we were unable to determine the characteristics of the hospitals in which individuals were treated. Medication is only one consideration; hospitals that use other techniques, such as bridge clinics to facilitate ongoing outpatient treatment, substance navigators and/or peer recovery coaches, and dedicated addiction consultation teams, may have more success linking patients to ongoing treatment and encouraging patients to start MOUD.^9,10,11^ There may also be reasons why patients do not want to start MOUD even when it is recommended, such as fear of precipitated withdrawal from buprenorphine^36^; recent opioid use, which would preclude naltrexone use; or when the patient is not yet ready for OUD treatment.^37^

### Limitations

There are several limitations to this study to consider. Primarily, this was a retrospective observational study that relied on administrative billing code data that captured hospital visits, medication dispensation, and outcomes in a single state. The Oregon APCD captures information on approximately 80% of the population, but 1 in 5 is not included. To determine outcomes, we required continuous enrollment with a maximum 60-day gap. In so doing, we likely excluded the most vulnerable patients who either did not have insurance or discontinued insurance during the study period. We considered certain basic patient characteristics but could not include others that may be important, such as location of residence (particularly rural vs urban, which can impact access to MOUD treatment), reason for hospital visit, or primary substance used (eg, prescribed opioids, heroin, or fentanyl). Although we attempted to consider severity of illness by including number of Elixhauser comorbidities, it is possible that only healthier patients, those who expressed interest in MOUD and motivation for recovery, or those who had another characteristic not available in our data received the treatment, which is a threat to validity. We were not able to ascertain individual comorbid conditions from our data. We could also not ascertain the readiness of patients for MOUD treatment, including whether it was offered but declined. We also did not determine the dosage of the MOUD given, whether treatment continued beyond the first 7 days, or whether the prescription was generated during or after the hospital visit.

## Conclusions

The findings of this cohort study suggest that initiation of MOUD after hospital visits is associated with reduced odds of opioid-related overdose at 6 months. Hospitals and their associated EDs should consider implementing programs and protocols to offer initiation of MOUD to patients with OUD who present for care.

## Notes

Supplement 1. **eTable 1.** Covariates associated with fatal or non-fatal overdose, focusing on the findings associated with receiving an opioid use disorder (OUD) formulation of buprenorphine 7 days post OUD-related hospitalization **eTable 2.** Covariates associated with fatal or non-fatal overdose, focusing on the findings associated with receiving an opioid use disorder (OUD) formulation of methadone 7 days post OUD-related hospitalization **eAppendix 1.** Medication for opioid use disorder codes **eAppendix 2.** Elixhauser comorbidity conditions **eAppendix 3.** Characteristics of included patients who were discharged after emergency department visit **eAppendix 4.** Characteristics of included patients who were discharged after hospitalization

Supplement 2.
Data Sharing Statement
