# Supplementary material for: Opioid Overdose After Medication for Opioid Use Disorder Initiation Following Hospitalization or ED Visit
Source: JAMA Netw Open. 2024 Jul 22;7(7):e2423954. doi: 10.1001/jamanetworkopen.2024.23954 (PMC11265135; doi:10.1001/jamanetworkopen.2024.23954)
Supplement: Supplement 1. — eTable 1. Covariates associated with fatal or non-fatal overdose, focusing on the findings associated with receiving an opioid use disorder (OUD) formulation of buprenorphine 7 days post OUD-related hospitalization eTable 2. Covariates associated with fatal or non-fatal overdose, focusing on the findings associated with receiving an opioid use disorder (OUD) formulation of methadone 7 days post OUD-related hospitalization eAppendix 1. Medication for opioid use disorder codes eAppendix 2. Elixhauser comorbidity conditions eAppendix 3. Characteristics of included patients who were discharged after emergency department visit eAppendix 4. Characteristics of included patients who were discharged after hospitalization [file jamanetwopen-e2423954-s001.pdf]

Supplemental Online Content

Weiner SG, Little K, Yoo J, et al. Opioid overdose after medication for opioid use disorder initiation following hospitalization or ED visit. *JAMA Netw Open.* 2024;7(7):e2423954. doi:10.1001/jamanetworkopen.2024.23954

eTable 1. Covariates associated with fatal or non-fatal overdose, focusing on the findings associated with receiving an opioid use disorder (OUD) formulation of buprenorphine 7 days post OUD-related hospitalization

eTable 2. Covariates associated with fatal or non-fatal overdose, focusing on the findings associated with receiving an opioid use disorder (OUD) formulation of methadone 7 days post OUD-related hospitalization

eAppendix 1. Medication for opioid use disorder codes

eAppendix 2. Elixhauser comorbidity conditions

eAppendix 3. Characteristics of included patients who were discharged after emergency department visit

eAppendix 4. Characteristics of included patients who were discharged after hospitalization

This supplemental material has been provided by the authors to give readers additional information about their work.

© 2024 Weiner SG et al. *JAMA Network Open.*

eTable 1. Covariates associated with fatal or non-fatal overdose, focusing on the findings associated with receiving an opioid use disorder (OUD) formulation of buprenorphine 7 days post OUD-related hospitalization.

	**Odds of overdose 6 months post OUD hospitalization**	**Odds of overdose 12 months post OUD hospitalization**
**Odds**	**aOR (95% CI)**	**aOR (95% CI)**
**Age 25-39 vs Less than 25**	0.83 (0.61 - 1.12)	0.74 (0.59 – 0.93)
**Age 40-54 vs Less than 25**	0.45 (0.31 – 0.64)	0.43 (0.33 – 0.57)
**Age 55-74 vs Less than 25**	0.39 (0.27 – 0.56)	0.40 (0.30 – 0.52)
**Age 75+ vs Less than 25**	0.22 (0.12 – 0.40)	0.18 (0.11 – 0.30)
**Sex Male vs Female**	1.50 (1.24 – 1.81)	1.55 (1.34 – 1.80)
**Medicaid vs Commercial**	1.54 (1.04 – 2.29)	1.72 (1.26 – 2.36)
**Insurance - Medicare vs Commercial**	1.14 (0.72 – 1.83)	1.01 (0.70 – 1.47)
**Insurance - Dual vs Commercial**	1.27 (0.76 – 2.12)	1.34 (0.90 – 2.00)
**Insurance - Unknown vs Commercial**	0.57 (0.22 – 1.52)	0.60 (0.28 – 1.30)
**Comorbidities 1-2 vs 0**	0.92 (0.70 - 1.21)	1.01 (0.81 – 1.25)
**Comorbidities 3+ vs 0**	1.06 (0.83 - 1.37)	1.16 (0.95 – 1.41)
**Received buprenorphine treatment in the 7 days post hospitalization vs none**	0.50 (0.27 – 0.95)	0.66 (0.43 – 1.02)

OUD = opioid use disorder. CI = confidence interval. aOR = adjusted odds ratio.

© 2024 Weiner SG et al. *JAMA Network Open.*

eTable 2. Covariates associated with fatal or non-fatal overdose, focusing on the findings associated with receiving an opioid use disorder (OUD) formulation of methadone 7 days post OUD-related hospitalization.

	**Odds of overdose 6 months post OUD hospitalization**	**Odds of overdose 12 months post OUD hospitalization**
**Odds**	**aOR (95% CI)**	**aOR (95% CI)**
**Age 25-39 vs Less than 25**	0.84 (0.62 - 1.14)	0.74 (0.59 - 0.94)
**Age 40-54 vs Less than 25**	0.46 (0.32 - 0.65)	0.44 (0.33 - 0.57)
**Age 55-74 vs Less than 25**	0.40 (0.27 - 0.58	0.40 (0.30 - 0.53)
**Age 75+ vs Less than 25**	0.23 (0.13 - 0.41	0.19 (0.11 - 0.30)
**effSex M vs F**	1.50 (1.23 - 1.82)	1.55 (1.34 - 1.80)
**Medicaid vs Commercial**	1.57 (1.06 - 2.33)	1.74 (1.27 - 2.38)
**Insurance - Medicare vs Commercial**	1.16 (0.73 - 1.86)	1.02 (0.71 - 1.49)
**Insurance - Dual vs Commercial**	1.29 (0.77 - 2.15)	1.36 (0.91 - 2.02)
**Insurance - Unknown vs Commercial**	0.59 (0.22 - 1.55)	0.61 (0.28 - 1.32)
**Comorbidities 1-2 vs 0**	0.93 (0.70 - 1.22)	1.02 (0.82 - 1.26)
**Comorbidities 3+ vs 0**	1.07 (0.83 - 1.38)	1.17 (0.96 - 1.42)
**Received methadone treatment in the 7 days post hospitalization vs none**	0.57 (0.28 - 1.17)	0.81 (0.51 - 1.30)

OUD = opioid use disorder. CI = confidence interval. aOR = adjusted odds ratio.

© 2024 Weiner SG et al. *JAMA Network Open.*

Appendix 1: Medication for opioid use disorder codes. Clinic administered codes are Healthcare Common Procedure Coding System (HCPCS) codes for administration of methadone (i.e. in an outpatient opioid treatment program), buprenorphine (including implantable and injectable formulations) and extended-release naltrexone. Outpatient pharmacy medications are determined by prescription drug monitoring program (PDMP) fills for formulations of buprenorphine that are indicated for the treatment of opioid use disorder, based on National Drug Code (NDC) value.

**Clinic Administered medication**	
Methadone	H0020 Alcohol And/Or Drug Services; Methadone Administration<br>H0033 Oral Medication Administration, direct observation
Buprenorphine	J0571 BUPRENORPHINE ORAL 1 MG<br>J0572 BUPRENORPHINE/NALOXONE ORAL </=TO 3 MG BPN<br>J0573 BUPRENORPHNE/NALOXONE ORAL >3 MG BUT </=6 MG BPN<br>J0574 BUPRENORPHINE/NLX ORAL >6 MG BUT </=TO 10 MG BPN<br>J0575 BUPRENORPHINE/NALOXONE ORAL >10 MG BUPRENORPHINE<br>J0570 Buprenorphine implant, 74.2 mg (e.g. Probuphine)<br>Q9991 Injection, buprenorphine extended-release (e.g. Sublocade), less than or equal to 100 mg<br>Q9992 Injection, buprenorphine extended-release (e.g. Sublocade), greater than 100 mg
Extended-release naltrexone (e.g. Vivitrol)	J2315

Outpatient pharmacy medications

NDC	Name	Strength	formulation
00054017613	BUPRENORPHINE HCL	2 mg	TAB SUBL
00054017713	BUPRENORPHINE HCL	8 mg	TAB SUBL
00054018813	BUPRENORPHINE-NALOXONE	2 mg-0.5 mg	TAB SUBL
00054018913	BUPRENORPHINE-NALOXONE	8 mg-2 mg	TAB SUBL
00093537856	BUPRENORPHINE HCL	2 mg	TAB SUBL
00093537956	BUPRENORPHINE HCL	8 mg	TAB SUBL
00093572056	BUPRENORPHINE-NALOXONE	2 mg-0.5 mg	TAB SUBL
00093572156	BUPRENORPHINE-NALOXONE	8 mg-2 mg	TAB SUBL
00228315303	BUPRENORPHINE HCL	8 mg	TAB SUBL
00228315403	BUPRENORPHINE-NALOXONE	2 mg-0.5 mg	TAB SUBL
00228315503	BUPRENORPHINE-NALOXONE	8 mg-2 mg	TAB SUBL

© 2024 Weiner SG et al. *JAMA Network Open.*

	00228315603	BUPRENORPHINE HCL	2 mg	TAB SUBL
	00378092393	BUPRENORPHINE HCL	2 mg	TAB SUBL
	00378092493	BUPRENORPHINE HCL	8 mg	TAB SUBL
	00406192303	BUPRENORPHINE-NALOXONE	2 mg-0.5 mg	TAB SUBL
	00406192403	BUPRENORPHINE-NALOXONE	8 mg-2 mg	TAB SUBL
	12496120201	SUBOXONE	2 mg-0.5 mg	FILM
	12496120203	SUBOXONE	2 mg-0.5 mg	FILM
	12496120401	SUBOXONE	4 mg-1 mg	FILM
	12496120403	SUBOXONE	4 mg-1 mg	FILM
	12496120801	SUBOXONE	8 mg-2 mg	FILM
	12496120803	SUBOXONE	8 mg-2 mg	FILM
	12496121201	SUBOXONE	12 mg-3 mg	FILM
	12496121203	SUBOXONE	12 mg-3 mg	FILM
	12496127802	SUBUTEX	2 mg	TAB SUBL
	12496128302	SUBOXONE	2 mg-0.5 mg	TAB SUBL
	12496130602	SUBOXONE	8 mg-2 mg	TAB SUBL
	12496131002	SUBUTEX	8 mg	TAB SUBL
	16590066630	SUBOXONE	2 mg-0.5 mg	TAB SUBL
	35356000407	SUBOXONE	8 mg-2 mg	TAB SUBL
	35356000430	SUBOXONE	8 mg-2 mg	TAB SUBL
	35356055530	BUPRENORPHINE HCL	2 mg	TAB SUBL
	35356055630	BUPRENORPHINE HCL	8 mg	TAB SUBL
	42291017430	BUPRENORPHINE-NALOXONE	2 mg-0.5 mg	TAB SUBL
	42291017530	BUPRENORPHINE-NALOXONE	8 mg-2 mg	TAB SUBL
	43063018407	SUBOXONE	8 mg-2 mg	TAB SUBL
	43063018430	SUBOXONE	8 mg-2 mg	TAB SUBL
	49999039507	SUBOXONE	2 mg-0.5 mg	TAB SUBL
	49999039515	SUBOXONE	2 mg-0.5 mg	TAB SUBL
	49999039530	SUBOXONE	2 mg-0.5 mg	TAB SUBL
	49999063830	SUBUTEX	2 mg	TAB SUBL
	49999063930	SUBUTEX	8 mg	TAB SUBL
	50383092493	BUPRENORPHINE HCL	2 mg	TAB SUBL

© 2024 Weiner SG et al. *JAMA Network Open.*

	50383093093	BUPRENORPHINE HCL	8 mg	TAB SUBL
	52959030430	SUBOXONE	8 mg-2 mg	TAB SUBL
	52959074930	SUBOXONE	2 mg-0.5 mg	TAB SUBL
	54123091430	ZUBSOLV	1.4 mg-0.36 mg	TAB SUBL
	54123095730	ZUBSOLV	5.7 mg-1.4 mg	TAB SUBL
	54123098630	ZUBSOLV	8.6 mg-2.1 mg	TAB SUBL
	54569549600	SUBOXONE	2 mg-0.5 mg	TAB SUBL
	54569573900	SUBOXONE	8 mg-2 mg	TAB SUBL
	54569573901	SUBOXONE	8 mg-2 mg	TAB SUBL
	54569573902	SUBOXONE	8 mg-2 mg	TAB SUBL
	54569639900	SUBOXONE	8 mg-2 mg	FILM
	54569640800	BUPRENORPHINE-NALOXONE	8 mg-2 mg	TAB SUBL
	54868570700	SUBOXONE	8 mg-2 mg	TAB SUBL
	54868570701	SUBOXONE	8 mg-2 mg	TAB SUBL
	54868570702	SUBOXONE	8 mg-2 mg	TAB SUBL
	54868570703	SUBOXONE	8 mg-2 mg	TAB SUBL
	54868570704	SUBOXONE	8 mg-2 mg	TAB SUBL
	54868575000	SUBOXONE	2 mg-0.5 mg	TAB SUBL
	55045378403	SUBOXONE	8 mg-2 mg	TAB SUBL
	55700014730	SUBOXONE	8 mg-2 mg	FILM
	55700018430	BUPRENORPHINE-NALOXONE	2 mg-0.5 mg	TAB SUBL
	59385001201	BUNAVAIL	2.1 mg-0.3 mg	FILM
	59385001230	BUNAVAIL	2.1 mg-0.3 mg	FILM
	59385001401	BUNAVAIL	4.2 mg-0.7 mg	FILM
	59385001430	BUNAVAIL	4.2 mg-0.7 mg	FILM
	59385001601	BUNAVAIL	6.3 mg-1 mg	FILM
	59385001630	BUNAVAIL	6.3 mg-1 mg	FILM
	63629402801	SUBOXONE	2 mg-0.5 mg	TAB SUBL
	63629403401	SUBOXONE	8 mg-2 mg	TAB SUBL
	63629403402	SUBOXONE	8 mg-2 mg	TAB SUBL
	63629403403	SUBOXONE	8 mg-2 mg	TAB SUBL
	63874108403	SUBOXONE	8 mg-2 mg	TAB SUBL
	63874108503	SUBOXONE	2 mg-0.5 mg	TAB SUBL
	63874117303	SUBUTEX	8 mg	TAB SUBL

© 2024 Weiner SG et al. *JAMA Network Open.*

	65162041503	BUPRENORPHINE-NALOXONE	8 mg-2 mg	TAB SUBL
	65162041603	BUPRENORPHINE-NALOXONE	2 mg-0.5 mg	TAB SUBL
	66336001630	SUBOXONE	8 mg-2 mg	TAB SUBL
	68071138003	SUBOXONE	8 mg-2 mg	TAB SUBL
	68071151003	SUBOXONE	2 mg-0.5 mg	TAB SUBL
	68258299903	SUBOXONE	2 mg-0.5 mg	TAB SUBL
	68308020230	BUPRENORPHINE HCL	2 mg	TAB SUBL
	68308020830	BUPRENORPHINE HCL	8 mg	TAB SUBL

© 2024 Weiner SG et al. *JAMA Network Open.*

Appendix 2: Elixhauser comorbidity conditions

**Condition**
Acquired immune deficiency syndrome
Alcohol abuse
Deficiency anemias
Autoimmune conditions
Chronic blood loss anemia
Leukemia
Lymphoma
Metastatic cancer
Solid tumor without metastasis, in situ
Solid tumor without metastasis, malignant
Cerebrovascular disease
Coagulopathy
Dementia
Depression
Diabetes with chronic complications
Diabetes without chronic complications
Drug abuse
Heart failure
Hypertension, complicated
Hypertension, uncomplicated
Liver disease, mild
Liver disease, moderate to severe
Chronic pulmonary disease
Neurological disorders affecting movement
Other neurological disorders
Seizures and epilepsy
Obesity
Paralysis
Peripheral vascular disease
Psychoses
Pulmonary circulation disease
Renal failure, moderate
Renal failure, severe
Hypothyroidism
Other thyroid disorders
Peptic ulcer with bleeding
Valvular disease
Weight loss

© 2024 Weiner SG et al. *JAMA Network Open.*

Appendix 3. Characteristics of included patients who were discharged after emergency department visit. OUD treatment = evidence of clinical-administered buprenorphine, methadone or extended-release naltrexone, or prescription drug monitoring program evidence of a filled prescription for a formulation of buprenorphine indicated for opioid use disorder.

	**Total, N = 8,345**		**Received OUD treatment within 7 days of discharge**			
			**No**		**Yes**	
	**N**	**Column %**	**N**	**Row %**	**N**	**Row %**
Total	8,345		7,651	91.2	694	8.3
**Age categories**						
18-24	943	11.3	838	88.9	105	11.1
25-39	3,184	38.2	2820	88.6	364	11.4
40-54	1,784	21.4	1639	91.9	145	8.1
55-74	1,986	23.8	1911	96.2	75	3.8
75+	448	5.4	443	98.9	5	1.1
**Gender**						
Female	4,118	49.4	3797	92.2	321	7.8
Male	4,226	50.6	3853	91.2	373	8.8
**Payer**						
Commercial	568	6.8	508	89.4	60	10.6
Medicaid	5,469	65.5	4891	89.4	578	10.6
Medicare	1,544	18.5	1514	98.1	30	1.9
Dual	763	9.1	737	96.6	26	3.4
**Comorbidities**						
0	1,668	20.0	1471	88.2	197	11.8
1-2	2,399	28.7	2151	89.7	248	10.3
3+	4,278	51.3	4029	94.2	249	5.8

	**N**	**%**	**N**	**%**	**N**	**%**
**Fatal or non-fatal overdose within 6 months**	244	2.9	230	3.0	14	2.0
**Fatal or non-fatal overdose within 12 months**	423	5.1	388	5.1	35	5.0
**Fatal overdose within 6 months**	19	0.2	18	0.2	1	0.1
**Fatal overdose within 12 months**	31	0.4	29	0.4	2	0.3
**Non-fatal overdose within 6 months**	228	2.7	214	2.8	14	2.0
**Non-fatal overdose within 12 months**	397	4.8	363	4.7	34	4.9

© 2024 Weiner SG et al. *JAMA Network Open.*

Appendix 4. Characteristics of included patients who were discharged after hospitalization. OUD treatment = evidence of clinical-administered buprenorphine, methadone or extended-release naltrexone, or prescription drug monitoring program evidence of a filled prescription for a formulation of buprenorphine indicated for opioid use disorder.

	Total, N = 13,890		Received OUD treatment within 7 days of discharge			
			No		Yes	
	N	Column %	N	Row %	N	Row %
Total	13,890		13,400	96.5	491	3.5
**Age categories**						
18-24	573	4.1	518	90.4	55	9.6
25-39	2,373	17.1	2116	89.2	257	10.8
40-54	2,518	18.1	2419	96.1	99	3.9
55-74	6,235	44.9	6160	98.8	75	1.2
75+	2,191	15.8	2187	99.8	4	0.2
**Gender**						
Female	7,690	55.4	7383	96.0	307	4.0
Male	6,200	44.6	6017	97.1	183	2.9
**Payer**						
Commercial	1,141	8.2	1106	96.9	35	3.1
Medicaid	5,317	38.3	4913	92.4	404	7.6
Medicare	4,887	35.2	4861	99.5	26	0.5
Dual	1,811	13.0	1791	98.9	20	1.1
Unknown	734	5.3	729	99.3	5	0.7
**Comorbidities**						
0	2,958	21.3	2822	95.4	136	4.6
1-2	2,222	16.0	2059	92.7	163	7.3
3+	8,710	62.7	8519	97.8	191	2.2

	N	%	N	%	N	%
**Fatal or non-fatal overdose within 6 months**	208	1.5	200	1.5	8	1.6
**Fatal or non-fatal overdose within 12 months**	335	2.4	324	2.4	11	2.2
**Fatal overdose within 6 months**	27	0.2	26	0.2	1	0.2
**Fatal overdose within 12 months**	45	0.3	43	0.3	2	0.4
**Non-fatal overdose within 6 months**	183	1.3	176	1.3	7	1.4
**Non-fatal overdose within 12 months**	294	2.1	285	2.1	9	1.8
