# Supplementary material for: Opioid Overdose After Medication for Opioid Use Disorder Initiation Following Hospitalization or ED Visit
Source: JAMA Netw Open. 2024 Jul 22;7(7):e2423954. doi: 10.1001/jamanetworkopen.2024.23954 (PMC11265135; doi:10.1001/jamanetworkopen.2024.23954)
Supplement: Supplement 2. — Data Sharing Statement [file jamanetwopen-e2423954-s002.pdf]

Data Sharing Statement

Weiner. Opioid Overdose After Medication for Opioid Use Disorder Initiation Following Hospitalization or ED Visit. *JAMA Netw Open*. Published July 22, 2024. doi:10.1001/jamanetworkopen.2024.23954

Data
Data available: No

Additional Information
Explanation for why data not available: The data used for this study cannot be shared by the authors due to state statute.
